# Ununited Fractures

**Published:** 1889-11-09

**Authors:** 


### SURGERY.

### UNUNITED FRACTURES.

The treatment of ununited intra-capsular fracture of the neck of the femur has always been most unsatisfactory, and a special interest attaches to a case reported not long ago by Dr. Nigrisoli, in its having been operated on with complete success by the late lamented Prof. Loreta. It is that of a man thirty-six years of age, who came under his care nineteen months after the accident. The limb was greatly wasted, the gluteal region flattened, and the fold of the buttock almost disappeared. Flexion and extension of the thigh were quite impossible, abduction and adduction extremely limited, and rotation gave great pain. The patient complained, too, of spasmodic jerks causing acute suffering. Loreta laid open the capsule, and after scraping away the fibrous connections and exposing the bony surfaces the fragments, placed them in apposition, having previously interposed a bundle of ten metallic threads, the ends of the wires being left protruding at the lower angle of the incision in the skin. The wound having been well irrigated, a drainage tube placed in position, and deep and superficial sutures applied, the whole was covered with sublimate dressings, and the limb fixed immovably by a long lateral splint. After five days the wires were withdrawn. The patient made a good recovery and was discharged on the fifty-first day perfectly cured.

